# Increasing incidence and prevalence of biochemically confirmed primary hyperparathyroidism in Stockholm, 2006-2020

**DOI:** 10.1210/clinem/dgag162

**Published:** 2026-04-11

**Authors:** David Thorsteinsson, Fredrik Granath, Robert Bränström, Jan Zedenius, Juan-Jesus Carrero, Inga-Lena Nilsson

**Affiliations:** Department of Breast, Endocrine Tumors and Sarcoma, Karolinska University Hospital, Stockholm 171 76, Sweden; Department of Molecular Medicine and Surgery, Karolinska Institutet, Stockholm 171 76, Sweden; Department of Medicine Solna, Division of Clinical Epidemiology, Karolinska Institutet, Stockholm 171 77, Sweden; Department of Breast, Endocrine Tumors and Sarcoma, Karolinska University Hospital, Stockholm 171 76, Sweden; Department of Molecular Medicine and Surgery, Karolinska Institutet, Stockholm 171 76, Sweden; Department of Breast, Endocrine Tumors and Sarcoma, Karolinska University Hospital, Stockholm 171 76, Sweden; Department of Molecular Medicine and Surgery, Karolinska Institutet, Stockholm 171 76, Sweden; Department of Medical Epidemiology and Biostatistics, Karolinska Institutet, Stockholm 171 77, Sweden; Division of Nephrology, Department of Clinical Sciences, Karolinska Institutet, Danderyd Hospital, Stockholm 182 88, Sweden; Department of Breast, Endocrine Tumors and Sarcoma, Karolinska University Hospital, Stockholm 171 76, Sweden; Department of Molecular Medicine and Surgery, Karolinska Institutet, Stockholm 171 76, Sweden

**Keywords:** hyperparathyroidism, incidence, prevalence, parathyroidectomy, epidemiology, biochemistry, parathyroid hormone, calcium

## Abstract

**Context:**

Primary hyperparathyroidism (PHPT) prevalence appears to be rising, but substantial underdiagnosis persists. Accurate epidemiological data are needed, requiring population-based studies using strict biochemical criteria.

**Objective:**

To determine incidence and prevalence of biochemically confirmed PHPT in Stockholm and characterize clinical recognition and surgical management.

**Methods:**

This is a population-based healthcare cohort study using prespecified biochemical diagnostic algorithms in the Stockholm Region, Sweden, 2006-2020, using the Stockholm CREAtinine Measurements database which covers two-thirds of the adult population, including ≥90% of individuals ≥65 years. Adults aged ≥20 years with paired calcium and parathyroid hormone (PTH) measurements meeting biochemical criteria for PHPT were included. Individuals with estimated glomerular filtration rate ≤30 mL/min/1.73 m^2^, secondary hyperparathyroidism, or prior parathyroid surgery were excluded. Main outcome measures were the annual incidence rate and point prevalence of biochemically confirmed PHPT.

**Results:**

Among 176 780 individuals contributing 578 227 PTH measurements, 10 190 patients fulfilling biochemical PHPT criteria were identified. Age- and sex-adjusted incidence rate increased from 2.81 (95% CI, 2.55-3.09) to 4.28 (95% CI, 3.98-4.59) per 10 000 person-years between 2009 and 2020, with the steepest increase among women aged ≥70 years. Prevalence increased from 0.35 to 4.76 per 1000 individuals. Median ionized calcium was 1.39 mmol/L. Clinical diagnosis of PHPT was documented in 5346 (52%) patients, of which 2800 (52%) underwent parathyroidectomy, with surgery rates stabilizing after 2013 at 4% to 5% annually.

**Conclusion:**

Using a large healthcare database, we observed substantially increasing PHPT incidence and prevalence, predominantly in older individuals. Only 52% received clinical diagnosis, underscoring the need for studies evaluating the long-term clinical burden of undiagnosed PHPT.

Primary hyperparathyroidism (PHPT) is a common endocrine disorder defined by disruption of the normal homeostatic relationship between circulating parathyroid hormone (PTH) and calcium. It is most frequently caused by autonomous PTH secretion from a single parathyroid adenoma. Multiglandular parathyroid disease occurs in about 10% to 15% and is more often associated with hereditary syndromes and young age of onset. Parathyroid carcinoma is an extremely rare cause of PHPT (1). The PHPT diagnosis is based on biochemical criteria, and contemporary classification is based on calcium levels and target-organ involvement, ranging from “classical” hypercalcemic disease with renal and/or skeletal manifestations to asymptomatic hypercalcemia. More recently, a normocalcemic variant has been described, characterized by elevated PTH despite normal calcium concentrations (2, 3). Parathyroidectomy is the only curative treatment for PHPT and has demonstrated benefit in more advanced disease, but its effectiveness remains controversial for milder forms without direct, measurable end-organ damage (4).

Advances in biochemical screening have increased detection of subtle abnormalities in the calcium–PTH axis, while overt skeletal and renal manifestations have become less frequent. The prevalence of this more indolent, mild phenotype appears to be rising in both Europe and the United States over the past 2 decades (5-7). At the same time, several studies have demonstrated substantial underdiagnosis of PHPT (8-11), further contributing to uncertainty regarding disease prevalence and healthcare resource requirements. In a recent nationwide study, we observed 6-fold variation in parathyroidectomy rates between Swedish regions (12). Patients from low-surgery regions had larger adenomas, higher calcium levels, and greater prevalence of kidney stones, osteoporosis, and hypertension at the time of surgery, suggesting that regional variation reflects differences in clinical detection thresholds and referral practices rather than true disease prevalence.

To address these uncertainties, robust population-based studies are needed that apply strict biochemical inclusion criteria, using calcium and PTH measurements, to capture both mild and classical forms of PHPT. Such an approach enables identification of patients managed in primary or tertiary care, as well as individuals with previously undiagnosed disease. The primary objective of this study was to determine the annual incidence rate and point prevalence of PHPT in the Stockholm Region using a population-based healthcare database. Secondary objectives were to quantify the proportion of patients with clinically PHPT diagnosis and the proportion undergoing parathyroidectomy.

## Materials and methods

### Study design

This is a register-based cohort study using the Stockholm CREAtinine Measurements (SCREAM) database (13, 14) to identify patients with biochemically diagnosed PHPT.

### Setting

The study was conducted in Stockholm Region, Sweden, which has approximately 2.2 million inhabitants served by a tax-funded universal healthcare system with 3 main laboratory providers (Karolinska University Hospital, Unilabs, and Aleris/Synlab). The study population comprised all adults aged ≥20 years with residency in Stockholm Region who were registered in the SCREAM database and fulfilled biochemical criteria for PHPT between 1 January 2006 and 31 December 2020, with follow-up data available through 31 December 2021.

#### Ethics and data protection

Statistics Sweden replaced Swedish personal identification numbers with random identifiers before providing deidentified data to researchers. This approach preserved participant anonymity and permitted waiver of informed consent. The Stockholm Regional Ethics Committee approved the SCREAM project (Dnr. 2017/793-31), with subsequent amendments approved by the Swedish Ethical Review Authority (Dnr. 2020-06719 and 2021-03366). A separate application for the present study received ethical approval from the Swedish Ethical Review Authority (Dnr. 2024-02555-01).

### Participants and biochemical case ascertainment

**Figure 1:**
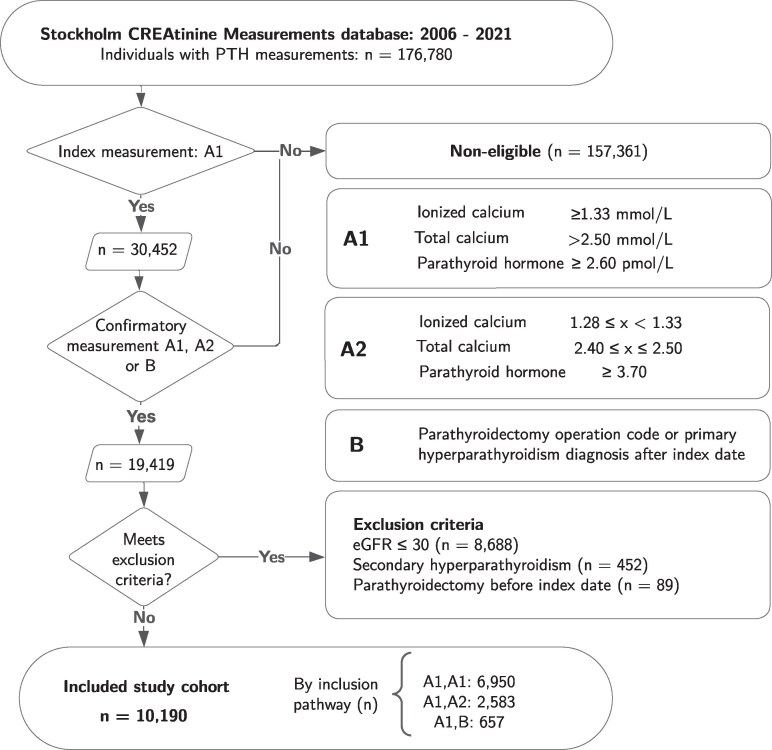
Flowchart detailing participant eligibility assessment, inclusion and exclusion procedures, and assembly of the final study cohort, including the diagnostic pathways used to define PHPT.

All individuals aged 20 years or older with a documented address in the Stockholm Region for at least 3 years before study entry were eligible for inclusion (Fig. 1). Individuals were evaluated for PHPT if a PTH measurement was available together with a calcium measurement; calcium and PTH were considered paired if obtained within 3 months, reflecting routine clinical practice where elevated calcium prompts follow-up PTH testing. The median interval between paired measurements was 0 days, with 75% of pairs obtained within 20 days and 90% within 50 days. When multiple qualifying paired measurements were available, we used the first qualifying pair. The index date was defined as the date of the first paired measurement meeting criterion A1 (Fig. 1).

Primary hyperparathyroidism is defined by high calcium levels with elevated or inappropriately normal PTH (15). The diagnostic algorithm in this study was prespecified and modeled on the population screening strategy of Lundgren et al (16), using calcium-stratified PTH thresholds: lower PTH thresholds for overt hypercalcemia (PTH ≥2.6 pmol/L) than for high-normal calcium (PTH ≥3.7 pmol/L). This approach reflects the principle that PTH should be suppressed in hypercalcemia, such that even low-normal PTH values are inappropriate when calcium is elevated. Biochemical or clinical confirmation at a separate timepoint was required to reduce transient misclassification.

Calcium and PTH were measured by th3ree laboratory providers in Stockholm Region. Total calcium reference intervals were standardized across all Stockholm laboratories in 2005 through the Nordic Reference Interval Project (upper limit 2.50 mmol/L), prior to the start of the study. For ionized calcium, the majority of measurements were reported by Karolinska University Laboratory and Synlab (91%), which shared a reference range of 1.15-1.33 mmol/L. A subgroup of Karolinska laboratories (Ersta Hospital) used a reference range of 1.08-1.32 mmol/L. Unilabs reported the remaining 9% of ionized calcium measurements with a reference range of 1.15 to 1.35 mmol/L. The corresponding proportions for total calcium measurements were 83% (Karolinska and Synlab) and 17% (Unilabs). We used 1.33 mmol/L as our primary ionized calcium threshold because this represented the upper limit for 91% of measurements. Normocalcemic proportions were reported based on each laboratory's own upper ionized calcium reference limit.

Parathyroid hormone was measured using both intact (second-generation) and whole (third-generation) assays with varying reference intervals: Karolinska used an intact assay (1.1-6.9 pmol/L) from 2006-2015 and then a whole PTH assay (1.6-6.0 pmol/L). Synlab used an intact assay (1.8-11 pmol/L). Unilabs used an intact assay (1.5-7.6 pmol/L). Current international guidelines interpret PTH qualitatively, as elevated or inappropriately normal relative to the prevailing calcium level, rather than defining an absolute diagnostic threshold (17). Both intact and whole PTH assays have similar diagnostic sensitivity for PHPT, and the precise PTH concentration is less relevant for diagnosis and management than the qualitative distinction from PTH-independent causes of hypercalcemia (18). Intact and whole PTH assays yield virtually identical results at PTH levels below ∼21 pmol/L in individuals with preserved kidney function (19). Full details of the laboratory instruments used for calcium and PTH measurements are provided in Table S1 (20). We defined 3 criteria: A1 required ionized calcium ≥1.33 mmol/L or total calcium >2.50 mmol/L and PTH ≥2.6 pmol/L; A2 required ionized calcium 1.28 to <1.33 mmol/L or total calcium 2.40 to 2.50 mmol/L and PTH ≥3.70 pmol/L. Criterion B was defined as a diagnostic code for PHPT or parathyroid adenoma (International Classification of Diseases 10th revision [ICD-10] E21 [excluding E21.1] or D35.1) or a parathyroidectomy procedure code (KVÅ BBA30) occurring at or after the index date.

Eligible individuals were included as incident PHPT cases if they met criterion A1 at the index measurement with confirmation by a subsequent paired measurement meeting A1 or A2 (pathways A1–A1 and A1–A2), or if they met criterion A1 at the index measurement followed by criterion B during follow-up (pathway A1–B). Patients with any history of kidney transplantation (KAS00-KAS20, KAS97) were excluded at the data extraction level.

Exclusion criteria were estimated glomerular filtration rate (eGFR) ≤30 mL/min/1.73 m^2^ within 180 days of either paired biochemical measurement, parathyroidectomy before the index date, or any history of secondary hyperparathyroidism diagnosis (ICD-10 E21.1).

### Variables

The primary outcomes were annual incidence rate and annual point prevalence of biochemically confirmed PHPT, defined according to the prespecified diagnostic algorithm (Fig. 1). For incidence rate analyses, the index date was the date specified by the algorithm as A1. Secondary outcomes were clinical recognition (PHPT or parathyroid adenoma diagnostic codes) and surgical treatment (parathyroidectomy procedure codes) recorded at or after the index date. Age at index date, sex, and calendar year were prespecified covariates used for stratified rate estimation and regression analyses. Laboratory variables included PTH (pmol/L), total calcium (mmol/L), serum ionized calcium (mmol/L), and creatinine (µmol/L).

Creatinine assays were standardized in Stockholm after 2010, and routine eGFR reporting was implemented across regional laboratories in 2016. To ensure consistency throughout the study period, all eGFR were calculated using the re-expressed Lund–Malmö Revised (r-LMR) equation, which has been validated in Swedish populations and serves as the standard reporting method in Stockholm since 2016 (21).

Parathyroid hormone was measured using validated immunoassays with platform-specific reference intervals (Table S1) (20). Values reported in ng/L were converted to pmol/L using a molecular weight of 9400 g/mol (1 pg/mL = 0.106 pmol/L). The diagnostic algorithm applied prespecified PTH thresholds of 2.6 pmol/L (Criterion A1) and 3.7 pmol/L (Criterion A2). As these thresholds fell within the lower range of reference intervals for both second- and third-generation assays, no additional standardization of PTH values was performed for case identification.

### Data sources

We used the SCREAM database, which links laboratory data to regional and national health registries using the Swedish personal identification number. SCREAM is a population-based database comprising all Stockholm residents, with laboratory data available for individuals who had at least one creatinine, cystatin C, or albuminuria measurement. SCREAM integrates 3 main data sources. Firstly, centralized laboratory data from Stockholm's 3 primary laboratory entities (Aleris/Synlab/Medilab, Karolinska University Hospital, and Unilabs) capture all measurements performed between 2006 and 2021, including timestamps, units, assay methods, and reference intervals. The SCREAM database provided continuous laboratory data throughout the study period, with the exception of December 2018, when data were unavailable. Secondly, the Stockholm Region healthcare administrative warehouse (Sw. Vårdanalysdatalager, VAL), covering all reimbursed healthcare services, provides diagnostic (ICD-10) and procedural (Swedish Procedure Coding System, KVÅ) codes from all publicly financed healthcare encounters since 1997, along with demographic data and migration dates enabling censoring for relocation outside Stockholm. Thirdly, national registries contribute vital status (Total Population Register), mortality data (Cause of Death Register), and cancer diagnoses (Swedish Cancer Register).

### Statistical analysis

Stratum-specific population counts obtained from Statistics Sweden (population as of 1 November of each year) were used to derive annual person-time denominators; person-years were approximated by the corresponding stratum population size for that year. Crude incidence rates were expressed as cases per 10 000 person-years. To account for temporal changes in population structure, incidence rates were directly standardized by age and sex to the 2013 Stockholm population using direct standardization with confidence intervals derived from the gamma distribution. Annual PTH measurement rates per 10 000 person-years were calculated using the same population denominator as the primary incidence analysis, restricted to individuals with eGFR ≥30 mL/min/1.73 m^2^ (r-LMR equation). When same-year creatinine measurements were unavailable, the most recent creatinine from the previous year was carried forward. Individuals were censored from first eGFR <30 mL/min/1.73 m^2^ onward. Confidence intervals assumed a Poisson distribution. Sensitivity analyses were performed using alternative creatinine carry-forward windows (no carry-forward, 6-month, and 2-year windows) to assess the robustness of temporal trends to different eGFR ascertainment strategies.

Temporal trends and incidence rate ratios (IRRs) were estimated using generalized linear models with a Poisson distribution. Calendar year was modeled as a continuous variable and, in separate models, as 3-year intervals. Models included age group and sex as covariates, with log(person-years) used as the offset. The year 2009 and period 2009-2011 were designated as the reference categories, as the initial 3-year period (2006-2008) was expected to capture prevalent cases misclassified as incident due to the absence of prior laboratory data.

To ensure complete case ascertainment, individuals were excluded if their index date occurred in 2021, as confirmatory testing could extend beyond available follow-up. Individuals with earlier index dates were included regardless of whether confirmatory testing occurred in 2021. Sensitivity analyses separately restricted the allowable interval between the index and confirmatory measurement to within 1 year and within 3 years.

Annual point prevalence was calculated as the number of individuals alive and resident in the Stockholm Region on 31 December of each year who met PHPT criteria divided by the corresponding population count on 1 November. Individuals contributed to prevalence from the index date until censoring at death or emigration.

Parathyroidectomy rates were calculated as the number of operations per calendar year divided by the mean annual number of prevalent patients (the average of consecutive year-end counts). The denominator included prevalent patients at risk, censored for death, first migration, or parathyroidectomy. Sex-stratified rates were reported per 100 persons with 95% CIs determined with exact binomial method.

Continuous variables are reported as mean (SD) or median (Q1-Q3), and categorical variables as counts (percentages). Between-group comparisons used the Mann–Whitney *U* test for nonnormally distributed continuous variables or Welch's *t*-test for normally distributed variables, and the Pearson χ^2^ test for categorical variables. Data management, statistical analyses, and figures were conducted in R version 4.5.1 using RStudio (Posit Software, PBC, Boston, MA, USA) with the epiR (22) and tidyverse (23) packages.

## Results

A total of 176 780 individuals were identified in SCREAM, contributing 578 227 PTH measurements during 2006-2020. The age- and sex-adjusted rate of PTH testing increased from 2006 through 2011 and thereafter plateaued (Fig. S1) (20). Sensitivity analyses using different creatinine carry-forward windows (0-24 months) showed that temporal trends in testing rates were robust to eGFR ascertainment strategy (IRR range: 1.024-1.030, all *P* < .001; Fig. S2) (20).

Figure 1 demonstrates the selection of the study cohort of 10 190 patients fulfilling the biochemical definition of PHPT. Baseline characteristics and the distribution of calcium and PTH measurements are shown in Table 1. At the index measurement, the median ionized calcium concentration was 1.39 mmol/L (1.35-1.44), and the median PTH concentration was 7.68 pmol/L (5.39-10.58). Median ionized calcium was slightly higher in men than in women (1.39 [1.36-1.46] vs 1.38 [1.35-1.43] mmol/L; *P* < .001), whereas median PTH concentrations were similar between sexes (7.78 [5.19-11.10] vs 7.68 [5.50-10.40] pmol/L for men and women, respectively; *P* = .96). At confirmatory measurement, the median ionized calcium and PTH concentrations were 1.37 (1.33-1.42) mmol/L and 7.47 (5.39-10.27) pmol/L, respectively (Table 1). A total of 464 patients (4.5%) had ionized calcium at the upper reference limit (1.33 and 1.35 mmol/L depending on laboratory) and were normocalcemic by definition at inclusion. Among these, the majority had preserved renal function: 113 (24%) had eGFR ≥90 and 288 (62%) had eGFR 60-89 mL/min/1.73 m^2^. Sixty-three patients (14% of the normocalcemic subgroup; 0.6% of the total cohort) had concurrent eGFR 30-59 mL/min/1.73 m^2^ (Chronic kidney disease, CKD stages G3a-G3b).

**Table 1:** Baseline characteristics including total numbers by sex, age group distribution, calcium, and PTH concentrations at index and confirmatory measurements and proportions with clinical diagnosis codes and parathyroidectomy

Characteristic	Total	Women	Men	*P^f^*
Age, years (SD)	63 (15)	64 (14)	58 (16)	**<**.**001**
Counts, n (%)	10 190 (100%)	7854 (77%)	2336 (23%)	**<**.**001**
20-29	303 (3%)	131 (2%)	172 (7%)	
30-39	477 (5%)	265 (3%)	212 (9%)	
40-49	1034 (10%)	718 (9%)	316 (14%)	
50-59	2311 (23%)	1814 (23%)	497 (21%)	
60-69	2617 (26%)	2073 (26%)	544 (23%)	
70-79	2362 (23%)	1914 (24%)	448 (19%)	
80-89	978 (10%)	839 (11%)	139 (6%)	
90+	108 (1%)	100 (1%)	8 (0%)	
Index measurement, median (Q1-Q3)				
Ionized calcium*^c^*	1.39 (1.35-1.44)	1.38(1.35-1.43)	1.39 (1.36-1.46)	**<**.**001**
Total calcium*^d^*	2.56 (2.53-2.61)	2.57(2.53-2.61)	2.56 (2.53-2.60)	
Parathyroid hormone	7.68 (5.39-10.58)	7.68 (5.50-10.40)	7.78 (5.19-11.1)	.96
Confirmatory measurement, median (Q1-Q3)				
Ionized calcium	1.37 (1.33-1.42)	1.37 (1.33-1.42)	1.37 (1.33-1.43)	
Total calcium	2.51 (2.45-2.58)	2.51 (2.45-2.58)	2.50 (2.44-2.57)	
Parathyroid hormone	7.47 (5.39-10.27)	7.47 (5.50-10.30)	7.36 (5.19-10.40)	
Diagnostic and operation codes*^e^*, n (%)				
Primary hyperparathyroidism*^a^*	5346 (52%)	4190 (53%)	1156 (49%)	
Parathyroidectomy*^b^*	2800 (52%)	2172 (52%)	628 (54%)	

Karolinska laboratory: Ionized calcium: 1.15-1.33 mmol/L; total calcium: 2.15-2.50 mmol/L; intact PTH (2006-2015): 1.1-6.0 pmol/L; whole PTH (2016-2021): 1.6-6.9 pmol/L. Synlab: Ionized calcium: 1.15-1.33 mmol/L; total calcium: 2.15-2.50 mmol/L; intact PTH: 1.8-11 pmol/L. Unilabs: Ionized calcium: 1.15-1.35 mmol/L; total calcium: 2.18-2.55 mmol/L; intact PTH: 1.5-7.6 pmol/L.

^*a*^Denominator is total population overall and by sex.

^*b*^Denominator is subgroup with confirmed E21 diagnosis.

^*c*^Ionized calcium measurements (n = 6402; 63%).

^*d*^Total calcium measurements (n = 3788; 37%).

^*e*^ICD-10-E21.0 and KVÅ procedure code BBA10-99.

^*f*^ *P* values derived from Welch's *t*-test (age), χ^2^ test (age group distribution), and Mann–Whitney *U* test (ionised calcium and parathyroid hormone). *P* values in bold denote statistical significance (*P* < .05).

**Figure 2:**
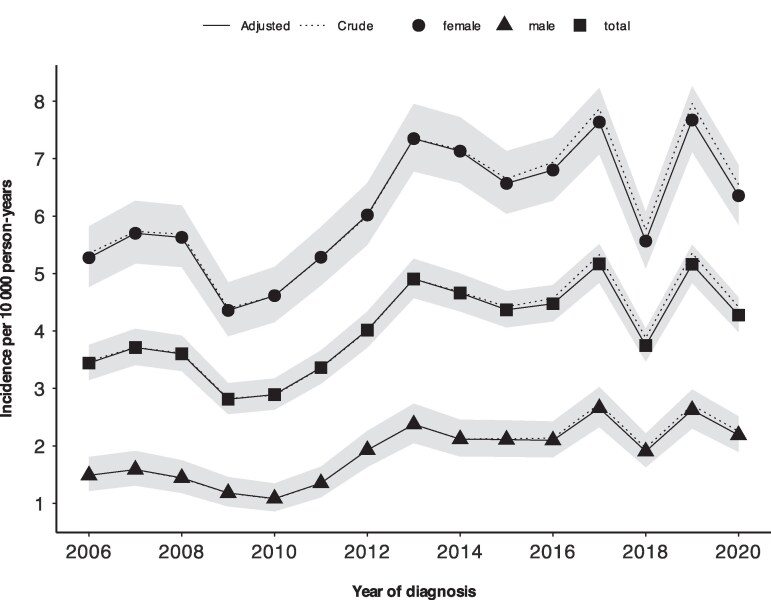
Adjusted and crude incidence rates are shown, with crude rates illustrated by a dashed line and 95% confidence intervals by gray ribbons. Marker shapes denote overall incidence (rectangles), women (circles), and men (triangles).

Crude and adjusted incidence rates of biochemically confirmed PHPT are shown in Fig. 2 (annual estimates in Table S2) (20). Following the 3-year run-in period, the overall age- and sex-adjusted incidence rate increased from 2.81 (95% CI, 2.55-3.09) per 10 000 person-years in 2009 to 4.28 (95% CI, 3.98-4.59) per 10 000 person-years in 2020.

**Figure 3:**
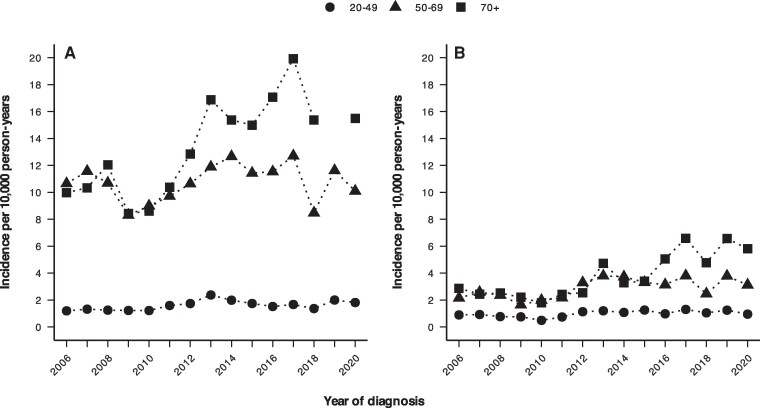
Crude incidence rates of primary hyperparathyroidism for the age groups 20-49 (circles), 50-69 (triangles), and ≥70 (rectangles) years across the study period. (A) Women; (B) men.

The incidence rate of PHPT increased significantly over time. Compared with the reference period 2009-2011, the adjusted IRR was 1.47 (95% CI, 1.38-1.57) in 2018-2020. When calendar year was modeled as a continuous variable, each year was associated with a 6% increase in incidence rate (IRR, 1.06; 95% CI, 1.05-1.06). The temporal increase in incidence rate varied substantially by age group, with patients aged ≥70 years exhibiting the steepest rise (Fig. 3). A significant interaction between age group and time period (*P* < .001) confirmed these age-specific differences. In continuous-year models, the annual increase was most pronounced among those aged ≥70 years (IRR per year, 1.10; 95% CI, 1.10-1.11), compared with more modest increases among those aged 50-69 years (IRR, 1.03; 95% CI, 1.03-1.03) and 20-49 years (IRR, 1.04; 95% CI, 1.03-1.04). Sensitivity analyses restricting the allowable interval between the index and confirmatory measurements to ≤1 year and ≤3 years yielded lower absolute incidence rate estimates but showed temporal patterns consistent with the main analysis supporting the robustness of the observed temporal trends (Fig. S3) (20).

**Figure 4:**
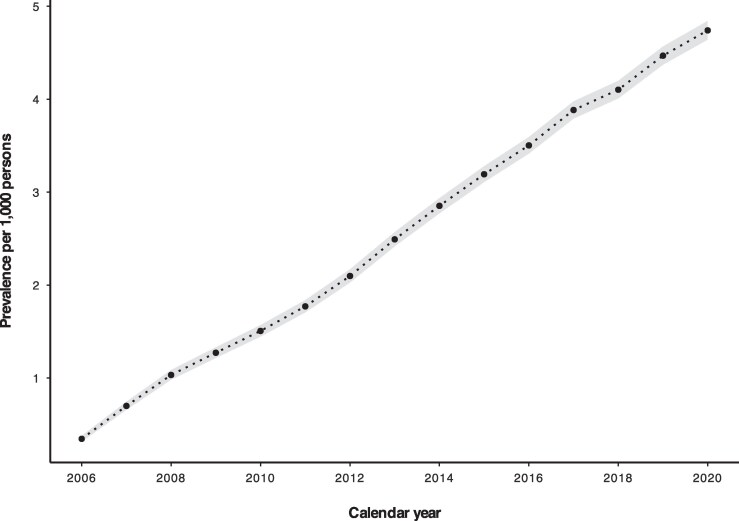
Annual point prevalence of primary hyperparathyroidism, based on individuals alive and resident at year-end, using the 1 November population as the denominator. Individuals were censored at death or migration.

Point prevalence increased from 0.35 per 1000 individuals at year-end 2006 to 4.76 per 1000 in 2020 (Fig. 4). Annual estimates with 95% confidence intervals, stratified by sex, are provided in Table S3 (20).

**Figure 5:**
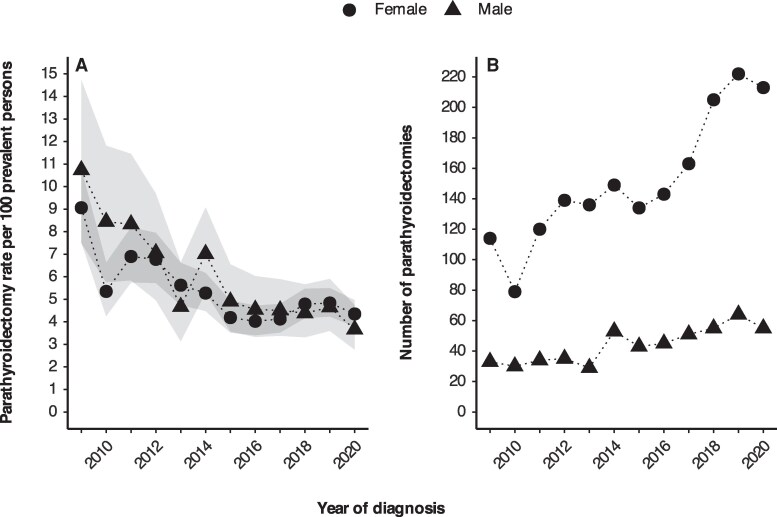
Parathyroidectomy patterns by sex. (A) Shows rates per 100 prevalent PHPT patients; (B) shows annual counts. Rates use the mean prevalence between consecutive year-ends as the denominator. Patients were included in the at-risk cohort during their year of surgery and censored thereafter. Rates and counts for women (circles) and men (rectangles) are shown separately. Shaded areas (A): 95% binomial confidence intervals.

Of all individuals meeting biochemical criteria for PHPT, 5346 (52%) received a clinical diagnosis, of whom 2800 (52%) underwent parathyroidectomy (Table 1). Median age at surgery was 60 years (interquartile range [IQR], 18) in women vs 58 years (IQR, 23) in men (*P* < .001). Although the absolute number of parathyroidectomies increased over the study period, the annual parathyroidectomy rate among those with prevalent PHPT decreased, stabilizing after 2013 at approximately 4% to 5% (Fig. 5).

## Discussion

This population-based cohort study identified more than 10 000 individuals with biochemically confirmed PHPT using prespecified diagnostic criteria and demonstrated a substantial increase in both incidence rate and prevalence in Stockholm between 2006 and 2020. Age- and sex-adjusted incidence rate rose from 2.81 to 4.28 per 10 000 person-years, with the steepest increase among women aged 70 years or older, whereas the incidence rate among younger individuals remained largely stable. Prevalence increased from 0.4 to 4.8 per 1000 persons. Most patients presented with mild biochemical disease (median ionized calcium of 1.39 mmol/L), with 25% of patients having ionized calcium concentrations >1.46 mmol/L at diagnosis. Despite biochemical confirmation, only 52% of cases had a recorded PHPT diagnosis, indicating that over half of the patients remain clinically undiagnosed. Despite rising disease prevalence, parathyroidectomy rates declined over time, stabilizing after 2013 at approximately 4% to 5% of the prevalent PHPT population annually.

Our incidence rate estimates from 2009 onward (2.81-4.28 per 10 000 person-years) are consistent with those reported in Tayside, Scotland (4-6 per 10 000 person-years), a comparable population served by a centralized healthcare system; however, incidence in Tayside appeared to stabilize over time, whereas it continued to rise in our cohort (5). The Kaiser Permanente Southern California study (1995-2010), which likewise defined PHPT using biochemical criteria, reported mean incidence rates of 6.6 per 10 000 person-years in women and 2.5 per 10 000 person-years in men, also concordant with our estimates. The somewhat lower starting point probably reflects the run-in period needed to establish the cohort in a database without prior laboratory history. While our use of ionized calcium and inclusion of normocalcemic patients may modestly increase ascertainment, our requirement for confirmatory biochemical testing increases diagnostic specificity but limits case identification compared with studies requiring only a single elevated measurement. Conversely, both Tayside and Kaiser Permanente studies relied on total calcium with thresholds above the upper reference limit, which would be expected to under ascertain milder PHPT (24).The convergence of incidence estimates across these independent datasets despite different ascertainment methods supports the validity of our findings. Also, prevalence patterns in our study similarly reflected the expected female-to-male ratio of approximately 3:1 (7.27 vs 2.23 per 1000 persons in 2020) with the highest incidence rates occurring in postmenopausal women, in keeping with prior reports (6, 7, 16, 25).

Our case ascertainment method reduces reliance on diagnostic codes and tertiary care referral patterns by employing biochemical algorithms based on calcium and PTH measurements during routine testing. However, ascertainment was conditioned on having a creatinine measurement, as both SCREAM inclusion and study eligibility required this test. Creatinine is typically ordered alongside calcium and PTH in clinical practice, and SCREAM coverage exceeds 90% among individuals aged 65 years or older, making it unlikely that this requirement materially affected ascertainment in the age groups with the greatest PHPT burden. For younger age groups, where SCREAM coverage is lower, this requirement may have resulted in modest underestimation; however, given the low incidence in these age groups, this is unlikely to have significantly affected overall population estimates.

Parathyroid hormone testing rates increased through 2012 and then plateaued. This changing diagnostic intensity may partly explain rising PHPT incidence rates in the first half of the study period, potentially reflecting enhanced case detection rather than a true increase in incidence. Incidence rate estimates in late follow-up were less stable due to incomplete laboratory capture in 2018 (data were missing for December) and the requirement for confirmatory testing, which precluded inclusion of individuals first meeting criteria in 2021. The COVID-19 pandemic may also have reduced testing intensity in 2020 because of restricted access to health care and deferred nonurgent evaluations. The introduction of the first national osteoporosis guidelines in Sweden in 2012 may have heightened awareness of calcium and PTH testing; however, a coordinated fracture liaison service was not implemented until after 2020. Increased diagnostic activity, broader access to biochemical testing, population aging, and evolving patterns of healthcare utilization are all likely to have contributed to the observed rise in incidence.

In a recent study, Kobylecki et al (26) showed that subtle biochemical changes within the upper end of the reference interval may represent early manifestations of emerging PHPT and could help identify individuals at increased risk for developing clinically overt disease. Given the risk of disease progression over time and the observed rise in both incidence rate and prevalence, the number of individuals requiring long-term follow-up is likely to increase substantially. As shown in our earlier work, Stockholm has comparatively high parathyroidectomy rates; therefore, the proportion of conservatively managed or undiagnosed patients in other regions of the country is likely even larger (12).

Of the 10 190 individuals meeting biochemical criteria for PHPT, 5346 (52%) had a clinical diagnosis. Among these clinically recognized patients, 2800 (52%) underwent parathyroidectomy during the study period. This 2-step selection from biochemical criteria to clinical diagnosis and then to surgical intervention may partly explain the regional variation we previously documented in parathyroidectomy rates across Sweden. That earlier analysis was limited to operated patients only and could not determine whether regional differences reflected variation in disease prevalence, clinical detection rates, or surgical selection thresholds. The current study addresses this limitation by providing population-based denominators for the first time, revealing that only half of biochemically defined PHPT receives clinical recognition, and among those recognized, about half proceed to surgery. Given the potential for disease progression, including development of osteoporosis and declining renal function in some patients, the growing burden of undiagnosed PHPT underscores the need for better clinical awareness and systematic follow-up protocols, particularly in elderly populations where disease prevalence is highest (27).

Minisola et al (28) and Bilezikian et al (29) have underscored the need to better define the long-term morbidity and mortality associated with mild PHPT, with or without parathyroidectomy, an area where contemporary evidence remains limited. As milder biochemical forms are increasingly recognized worldwide, there is a clear need for coordinated efforts to better define the epidemiology of both hypercalcemic and normocalcemic forms. At the same time, large population-based cohort studies are required to evaluate the long-term clinical and economic outcomes of surgical vs conservative management in this growing, predominantly older population with mild disease. Such studies should incorporate age, comorbidity, and baseline biochemical severity and assess a broad range of outcomes, including cardiovascular events, cognition, medication use, healthcare utilization, survival, and healthcare costs. This study cohort, with comprehensive biochemical data, national registry linkage, and near-complete follow-up, offers a strong foundation for addressing these critical knowledge gaps in the future.

These findings are most applicable to healthcare systems with broad access to biochemical testing. Sweden's low-barrier, tax-funded system likely increases diagnostic intensity, and absolute incidence and prevalence estimates may therefore exceed those in settings with higher cost barriers or less routine laboratory evaluation.

Swedish law prohibits recording ethnicity in healthcare databases. National data from Statistics Sweden indicate the proportion of foreign-born residents and Swedish-born individuals with 2 foreign-born parents increased from approximately 10% in 2006 to 20% in 2020, with immigrants predominantly of European origin. The study population can thus be assumed to be predominantly of Northern European descent. Given reported ethnic differences in PHPT incidence in US cohorts (7), our estimates may underestimate disease prevalence in more ethnically diverse populations.

### Strengths and limitations

The primary strength of this study is the ability to estimate PHPT incidence rates in a large, population-based cohort not subjected to selection bias from physician referrals or parathyroid surgery. This is evidenced by the finding that only half of the patients identified with biochemical criteria had received a diagnostic or procedural code for PHPT, highlighting the substantial underdiagnosis of this disease.

Several limitations warrant consideration. The SCREAM database captures approximately 67% of the adult population of Stockholm Region, with age-dependent coverage exceeding 90% among individuals aged 65 years or older (13, 14). Lower coverage in younger age groups reflects reduced healthcare utilization and consequently fewer opportunities for biochemical screening, which may lead to underestimation of PHPT incidence in these populations.

Certain conditions that biochemically mimic PHPT are unlikely to substantially affect our results. Our exclusion of patients with eGFR ≤30 mL/min/1.73 m^2^ (Kidney Disease Improving Global Outcomes, KDIGO stages G4-G5) was implemented to minimize misclassification from secondary hyperparathyroidism due to chronic kidney disease. Patients with mild to moderate renal insufficiency (KDIGO stages G3a and G3b) were retained, allowing capture of PHPT patients with renal complications. While this approach may have misclassified some cases of secondary hyperparathyroidism as PHPT, secondary hyperparathyroidism typically presents with normal or low-normal calcium concentrations. Our requirement for ionized calcium ≥1.33 mmol/L therefore substantially reduces this misclassification risk even among patients with moderate renal impairment. Conversely, while our eGFR threshold may have excluded some patients with true PHPT and advanced renal disease, including severe chronic kidney disease would have introduced a high proportion of secondary hyperparathyroidism cases, substantially reducing diagnostic specificity. Classification is more challenging, however, for the small proportion of patients included at the upper reference limit of ionized calcium (1.33 and 1.35 mmol/L, depending on laboratory), who are normocalcemic by definition. Among these 464 individuals, 63 (13.6%) had concurrent eGFR 30 to 59 mL/min/1.73 m^2^, where the possibility of secondary hyperparathyroidism contributing to PTH elevation cannot be excluded. Because vitamin D measurements were unavailable, residual misclassification related to vitamin D insufficiency in this subgroup also cannot be fully excluded. However, these 63 patients represent only 0.6% of the total cohort, and even under the conservative assumption that all were misclassified, the impact on overall incidence and prevalence estimates would be negligible. Secondary hyperparathyroidism due to vitamin D deficiency was not explicitly excluded; however, misclassification is improbable since this condition seldomly presents with elevated ionized calcium. Our PHPT definition required both abnormally elevated PTH and ionized calcium ≥1.33 mmol/L, well above levels generally observed with vitamin D deficiency in Nordic populations (30). Lithium-associated hyperparathyroidism was not excluded but is often considered a subgroup of PHPT. A recent nationwide study found that approximately 2% of Swedish patients undergoing parathyroid surgery had lithium exposure, compared with 0.5% in a reference population (31) suggesting minimal impact on our overall estimates. Familial hypocalciuric hypercalcemia may present with hypercalcemia and nonsuppressed PTH and could not be reliably distinguished in the absence of urinary calcium measurements. However, given its low estimated prevalence (approximately 1 in 78 000 in earlier estimates, with potentially higher rates suggested by recent genetic studies (32, 33)), any resulting misclassification is expected to have negligible effects on our incidence and prevalence estimates.

This population-based cohort of over 10 000 biochemically confirmed PHPT patients provides important opportunities for future research. Systematic case ascertainment not requiring clinical diagnosis captures the disease spectrum as it presents in clinical practice, including patients managed both surgically and conservatively. Linkage to Swedish national registries enables assessment of long-term clinical outcomes, healthcare utilization, and associated costs. Such data may help address current evidence gaps regarding optimal management strategies and inform evidence-based clinical guidelines, particularly for the growing elderly population affected by PHPT.

## Data Availability

Data are not available to other researchers due to ethical and legal restrictions. R code scripts can be provided via GitHub upon request.
